# Design and simulation of a tunable parity-time symmetric optoelectronic oscillator utilizing integrated components

**DOI:** 10.1038/s41598-024-67047-0

**Published:** 2024-07-11

**Authors:** Farnaz Ahmadfard, S. Esmail Hosseini

**Affiliations:** Department of Communications and Electronics, School of Electrical and Computer Engineering, Shiraz University, Shiraz, Iran

**Keywords:** Microwave photonics, Optics and photonics, Integrated optics

## Abstract

Non-Hermitian photonics, relaying on parity-time (PT) symmetry, have shown promise in achieving mode selection for optical or microwave single-mode oscillation. Typically, a PT-symmetric system is constructed using two coupled loops with identical geometry. This article utilizes the PT-symmetry property to select a single frequency mode in an optoelectronic oscillator (OEO). However, traditional OEO implementations often involve discrete components, limiting widespread adoption due to factors such as size, weight, power consumption, and cost. Our aim in this paper is to leverage integrated components within the OEO loop. The proposed structure incorporates an integrated micro-ring resonator (MRR) with a high-quality factor (Q-factor) that serves both as a modulator and a resonator. Additionally, we suggest employing an adjustable integrated power splitter utilizing a micro heater to balance the gain and loss of two mutually coupled OEO loops. In this configuration, two integrated photo detectors (PD) are also utilized. In this setup, the single-frequency mode can be easily identified by simultaneously utilizing the properties of PT-symmetry and an integrated high-Q-factor resonator, obviating the need for a narrowband microwave filter. By adjusting the center frequency of the microwave photonic filter (MPF), the frequency of the generated signal can be tuned over a wide range. For instance, setting the generated frequency of the microwave signal to 11.5 GHz results in a measured phase noise of − 76.5 dBc/Hz at a 10-kHz offset frequency, with a side mode suppression ratio (SMSR) of 40 dB.

## Introduction

Optoelectronic oscillators (OEOs) play a crucial role in generating microwave signals with minimal phase noise. An OEO combines microwave and photonic components within a feedback loop mechanism, finding applications in diverse domains such as wireless communication, radar systems, sensor networks, and advanced microwave systems^1–3^.

To achieve efficient OEOs characterized by lower phase noise, it is essential to employ OEO cavities with high quality factors (Q-factors). It should be noted that if a fiber delay line with a very long length is used to increase the Q-factor of the OEO loop, the eigenfrequency modes will be compressed together, and the free spectral range (FSR) will decrease^4^. Consequently, filtering a single oscillation mode becomes challenging, necessitating the use of a narrow-band filter. However, there are many challenges in achieving an electrical bandpass filter (EBF) with a narrow bandwidth^5–8^.

Various strategies have been proposed to address this challenge, including employing a tunable-bandwidth microwave photonic filter (MPF) as an optical notch filter. Examples include phase-shift fiber Bragg gratings (PS-FBGs)^9^or micro-ring resonators (MRR) with exceptionally high Q-factor^10^. However, this approach confines frequency mode selection to the bandwidth of the MPF; hence, the bandwidth of the selected frequency mode needs to be as narrow as possible.

Recently, a novel concept known as parity time symmetry (PT-symmetry), has been proposed as a technique for selecting modes in a single-mode lasers^11–13^ and electrical oscillators^14^. In a PT-symmetry system, two coupled feedback loops are necessary, which are geometrically similar but with one exhibiting gain and the other exhibiting loss. When the gain and loss coefficients are equal and exceed the coupling coefficient, PT-symmetry is broken resulting in a dominant mode that has a significantly different gain compared to other modes. Consequently, this technique enables the achievement of single-mode oscillation without the need for a narrow-band filter^15–20^.

In our study, we propose an integrated OEO with PT- symmetry. This innovative setup combines a high-Q-factor MRR that serves both as a modulator and a frequency selector. To enhance control over the system, we've introduced a versatile integrated power splitter comprising two Multi-Mode Interferences (MMIs) and two straight waveguides with equal lengths. By integrating a micro heater on one of the waveguides, we can manipulate the phase difference between the outputs, thereby managing gain and loss within the loops and achieving a single frequency mode more effectively. This control mechanism facilitates the achievement of a single frequency mode. In this configuration, two integrated photodetectors (PDs) are used, significantly reducing the size of the PT-symmetric OEO. In the OEO cavity, an MRR-based MPF is integrated to fine-tune the oscillation frequency of the OEO.

By utilizing the high-Q-factor MRR and PT symmetry properties in our design, we simplify mode selection and enhance the tunability of the OEO. Our setup generates a microwave signal that can be tuned from 0 to 20 GHz by adjusting the center frequency of the MPF. When the generated microwave signal frequencies are tuned to 6.2 GHz and 11.5 GHz, the measured single sideband (SSB) phase noise at a 10-kHz offset frequency is − 79.22 dBc/Hz and − 76.5 dBc/Hz, respectively, with an impressive side mode suppression ratio (SMSR) of 40 dB.

## Operation principle

**Figure 1:**
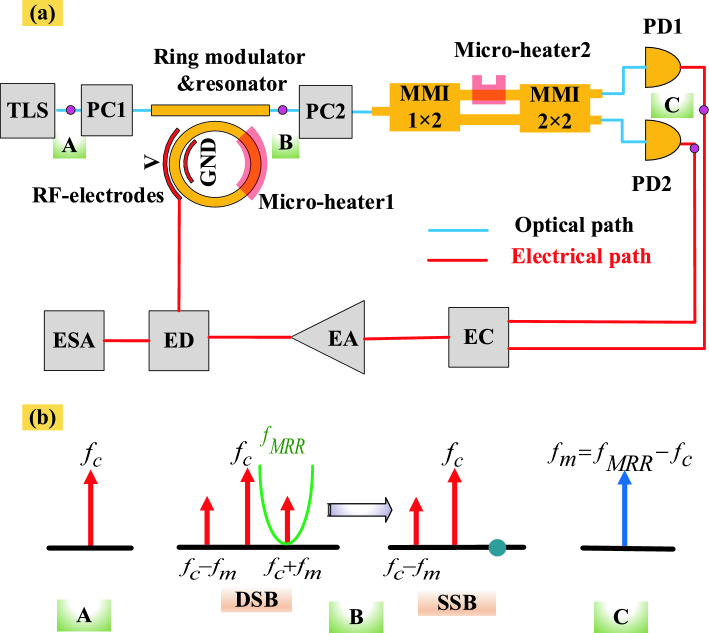
(**a**) schematic diagram of the proposed integrated PT-Symmetric OEO includes the following components: TLS (Tunable Laser Source), PC (Polarization Controller), MMI (Multimode interference), PD (Photodetector), EC (Electrical Combiner), EA (Electrical Amplifier), ED (Electrical Divider), and ESA (Electrical Spectrum Analyzer), (**b**) Working principle of the proposed OEO in different locations.

In the proposed optical architecture illustrated in Fig. 1a, a tunable PT-symmetric OEO utilizing integrated components is depicted. An integrated tunable MPF has been implemented, enabling mode selection and providing enhanced tunability. As shown, a tunable laser source (TLS) emits light directed into a micro-ring, which functions as both a modulator and a resonator. A polarization controller (PC1) adjusts the state of polarization (SOP) of the optical carrier.

To enable the micro-ring to serve as both a resonator and a modulator, its structure includes RF electrodes and a micro-heater. By applying a bias voltage to the RF electrodes, modulation is achieved. Additionally, the resonance frequency of the MRR can be adjusted using the micro-heater.

As illustrated in Fig. 1b, at point A, an optical signal with a frequency $f_{c}$ is generated. When this signal passes through the micro-ring and a bias voltage is applied to the RF electrodes, the carrier signal is modulated, creating an optical double sideband (ODSB) signal. At point B, by applying electrical power to the micro-heater1 located above the micro-ring, the ring resonator acts as a notch filter and the resonance frequency can be adjusted to align with $f_{c} + f_{m}$, converting the ODSB signal to an optical single sideband (OSSB). This process allows the high-Q MRR to transform the phase-modulated signal into an intensity-modulated signal (PM to IM conversion) during transmission. Incorporating a high-Q-factor resonator increases the Q-factor of the overall OEO system.

Subsequently, another polarization controller (PC2) adjusts the SOP of the modulated signal. The signal then enters a MMI device that splits the optical power evenly with a 50:50 ratio. Then the two outputs of the MMI 1 × 2 are connected to two straight waveguides with equal lengths. A micro-heater2 in the upper waveguide adjusts the power distribution between the two outputs. Following this, the MMI 2 × 2 converts the optical signals into microwave frequencies, at point C, which are detected by a photodetector (PD). The PD sends these microwave signals to an electrical combiner (EC), which merges them. The combined signal is then amplified by electrical amplifiers (EAs) and fed back into the ring modulator, completing the OEO loop.

If we examine the impact of each component in the proposed structure as a whole, we will see the following:

The TLS is crucial for determining the initial frequency and stability of the optical signal. Its stability and tuning accuracy directly influence the overall frequency stability and noise performance of the OEO. Any fluctuations or instability in the TLS will propagate through the system, affecting the microwave signal quality. A PC1 adjusts the SOP of the optical carrier, ensuring proper alignment before modulation. The MRR, acting as both a modulator and resonator, ensures narrow resonance linewidths vital for efficient modulation and selective filtering, which affects the signal-to-noise ratio (SNR) and purity of the microwave signal. The MRR’s performance in terms of insertion loss and modulation depth significantly impacts the overall efficiency of the OEO. The micro heater1 adjusts the resonance frequency of the MRR to align with the desired frequency, converting the signal from ODSB to OSSB, and ensuring single-mode operation. Subsequently, another PC2 adjusts the SOP of the modulated signal. The MMI 1 × 2 with a micro heater2 is critical for mode selection and phase adjustment. Precise control over the splitting ratio and minimal excess loss are necessary for accurate signal manipulation. The micro heater2 allows for fine temperature control, adjusting the phase and helping maintain coherence and stability in the loop, playing a key role in preventing mode hopping and ensuring single-mode operation.

The straight waveguide's low propagation loss and minimal dispersion are essential for preserving the quality of the optical signal as it travels through the system, ensuring the signal remains strong and undistorted, critical for maintaining the integrity of the microwave signal generated by the OEO.

The MMI 2 × 2 component is responsible for efficient coupling and splitting of the optical signals to the photodetectors. Its performance in minimizing crosstalk and loss is vital for ensuring that the optical signals reaching the photodetectors are of high quality and properly phased, necessary for accurate optical-to-electrical conversion.

Photodetectors convert optical signals to electrical signals. Their high responsivity, low noise, and fast response times are crucial for achieving a high SNR in the microwave domain. Any noise or inefficiency in the photodetectors will degrade the overall performance and quality of the microwave signal.

The combiner integrates the microwave signals from the photodetectors with minimal loss and phase shift. Efficient combination is essential for producing a strong and coherent microwave signal, crucial for stable oscillation in the OEO. The amplifier boosts the microwave signal to a usable level, with high gain, low noise figure, and broad bandwidth necessary to amplify the signal without introducing significant noise. The amplifier's performance directly affects the signal quality and stability, impacting the overall efficiency and effectiveness of the OEO.

The divider splits the amplified microwave signal into two parts with minimal loss and phase distortion. Accurate division ensures that the feedback signal to the modulator has the correct amplitude and phase, crucial for maintaining stable oscillations and overall system performance.

The Electrical Spectrum Analyzer (ESA) measures the characteristics of the microwave signal with high resolution and sensitivity, providing critical feedback on the signal quality, allowing for adjustments and optimization of the OEO. The performance of the ESA influences the ability to accurately assess and fine-tune the system.

The key components that make up the MPF in the OEO system are listed here. Firstly, high-Q MRR that is used for initial mode selection and precise tuning of the oscillation frequency. Besides the (Mach–Zehnder Interferometer) MZI is used for precise power splitting between the outputs of the 2 × 2 MMI. A microheater is placed on one of the MZI branches to accurately control gain and loss of two loops in the structure. By adjusting the voltage applied to the microheater on one of the MZI branches, the gain and loss of the loops can be precisely controlled, improving mode selection. High-speed silicon–germanium photodetectors are used to convert optical signals into electrical signals. These photodetectors are employed to recover microwave signals from the MMI outputs. Two identical photodetectors are used to accurately recover microwave signals and improve the SMSR. Microheaters are used for fine-tuning the resonance frequency of the MRR and controlling the gain and loss in the MZI structure. By applying voltage to the microheaters, thermal changes are induced, causing changes in the refractive index, which in turn tunes the resonance frequency and the power splitting ratio.

These components play a crucial role in fine-tuning the oscillation frequency, enhancing mode selection, and reducing phase noise, significantly boosting the overall performance of the OEO system. Each element in the proposed OEO structure is essential for maintaining the system's performance and stability. Their integration and optimization guarantee efficient and stable operation, high signal quality, and minimized noise, all of which are vital for the effective functioning of the OEO.

In this design, the implementation of PT-symmetry plays a crucial role in achieving single-mode oscillation without the need for a narrow-band microwave filter. PT-symmetry is based on the principle that non-Hermitian systems can exhibit real eigenvalues with non-orthogonal eigenstates. As the system approaches the critical point of PT-symmetry breaking, it transitions from real to complex eigenvalues, a phenomenon that occurs as the non-Hermitian parameter increases^21–23^.

In practical applications within photonics and electronics, a PT-symmetric system utilizes two identical feedback loops, one with gain and the other with an equivalent loss. This configuration is highly effective in isolating a single oscillation mode, eliminating the need for additional mode-selection components^24–27^.

As illustrated in Fig. 1, proposed OEO structure incorporates two interconnected feedback loops. The micro heater situated in the upper arm of the waveguide is strategically employed to finely balance the gain and loss in these loops, facilitating the maintenance of PT-symmetry and thereby promoting stable single-mode operation.

Theoretically, the behavior of the n-th oscillation mode in the two coupled loops can be described by a set of coupled differential equations as follows^16^:

1 $$\frac{{da_{n}^{(1)} }}{dt} = \left[ {j\Delta \omega_{n}^{(1)} + g} \right]a_{n}^{(1)} - j\mu a_{n}^{(2)}$$

2 $$\frac{{da_{n}^{(2)} }}{dt} = \left[ {j\Delta \omega_{n}^{(2)} + \gamma } \right]a_{n}^{(2)} - j\mu a_{n}^{(1)}$$

In the equations mentioned above, $a_{n}^{(1)}$ and $a_{n}^{(2)}$ denote the amplitudes of the nth oscillation modes in each respective loop. The terms $g$ and $\gamma$ represent the gain and loss within each loop. $\mu$ is the coupling coefficient between the two loops. Additionally, $\omega_{n}^{(1,2)}$ refers to the original resonance frequencies in each loop, while $\Delta \omega_{n}^{(1,2)} = \omega_{n} - \omega_{n}^{(1,2)}$ indicates the frequencies that have been retuned in each loop.

By solving Eqs. (1) and (2), the eigenfrequencies of the nth oscillation mode within the OEO loop can be determined as follows

3 $$\omega_{n \pm } = \frac{{\omega_{n}^{(1)} + \omega_{n}^{(2)} }}{2} + \frac{j(g - \gamma )}{2} \pm \sqrt {\mu^{2} - \left( {\frac{g + \gamma }{2} - \frac{{j\left[ {\omega_{n}^{(1)} - \omega_{n}^{(2)} } \right]}}{2}} \right)^{2} } .$$

If the lengths of the coupled loops are equal, then it can be concluded that $\omega_{n}^{(1)} = \omega_{n}^{(2)}$. Furthermore, considering the PT symmetry, the gain of a loop will be equal to the magnitude of the loss in another loop i.e., $g = \gamma$. Therefore, relation (3) will be simplified as follows:

4 $$\omega_{n \pm } = \omega_{n} \pm \sqrt {\mu^{2} - \gamma^{2} }$$

**Figure 2:**
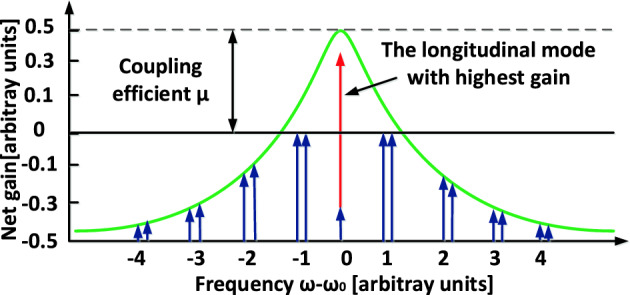
The process of selecting modes based on the principle of the PT-symmetric OEO.

In the equation discussed, $\Delta = \sqrt {\mu^{2} - \gamma^{2} }$ represents the complex detuning of the two PT-supermodes relative to the central frequency $\omega_{n \pm }$. Consequently, when $\left( {\gamma < \mu } \right)$ is real, it results in two real roots for $\Delta$. This indicates that the PT symmetry of the nth mode is preserved. However, if $\left( {\gamma > \mu } \right)$ becomes imaginary, this signifies a breaking of the PT symmetry for the nth mode, leading to the formation of amplified and attenuated modes within each loop. Under these conditions, the amplified mode experiences significantly more gain compared to other modes, facilitating stable single-mode oscillation as illustrated in Fig. 2.

## Simulation results and discussion

### Simulation of an integrated ring modulator /resonator, a tunble optical power spilitter and a PD

In this design, the realization of the OEO involves integrating several key components as shown in Fig. 1. These include the ring modulator/resonator, a tunable optical power splitter, and a PD.

**Figure 3:**
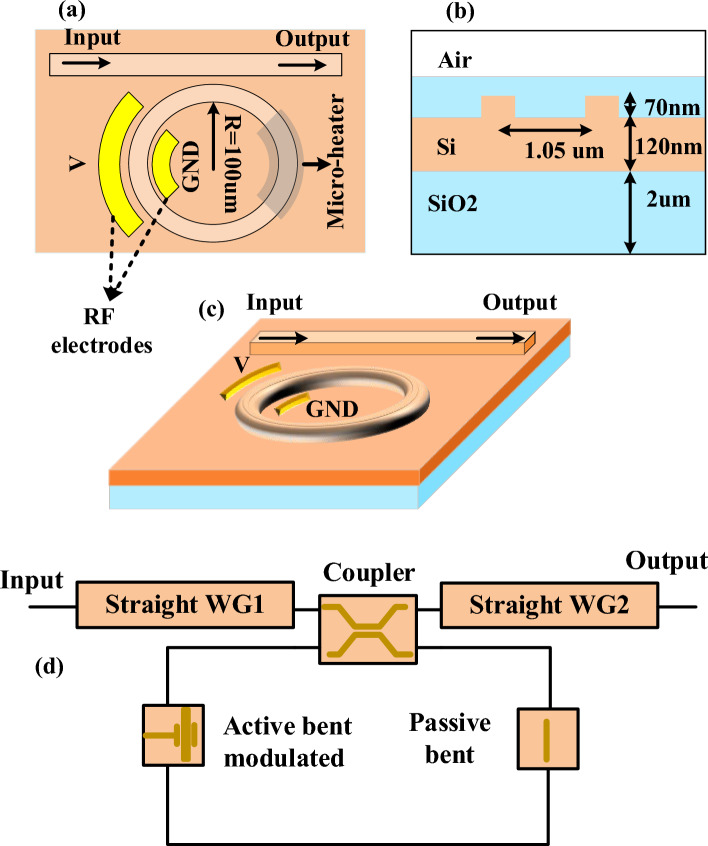
The simulated MRR is depicted in various views: (**a**) the top view, (**b**) the cross-section of the coupling area, (**c**) the perspective view, and (**d**) Diagrammatic representations of the simulated ring modulator and its subcomponents without applying electrical power to the micro heater.

Initially, our simulation focuses on the ring modulator/resonator as depicted in Fig. 3a–c illustrates the schematic configuration of a high Q-factor MRR in various views. The radius (R) of the MRR is set to a specific value 100 μm. To meet the requirement for a high Q-factor, the dimensions of the ring resonator need to be substantial. However, simulating large structures with the finite difference time domain (FDTD) method can be computationally intensive and time-consuming. Instead, we employ the interconnect method, which uses specialized solvers for mode propagation analysis to simulate integrated photonic circuits. To streamline the simulation process, we deconstruct the ring modulator structure shown in Fig. 3d into its individual components, such as directional couplers and both straight and bent waveguides. We then independently verify the performance of these components.

Subsequently, these elements are integrated into a circuit configuration similar to that illustrated in Fig. 3d, allowing for an accurate simulation of the entire device.

**Figure 4:**
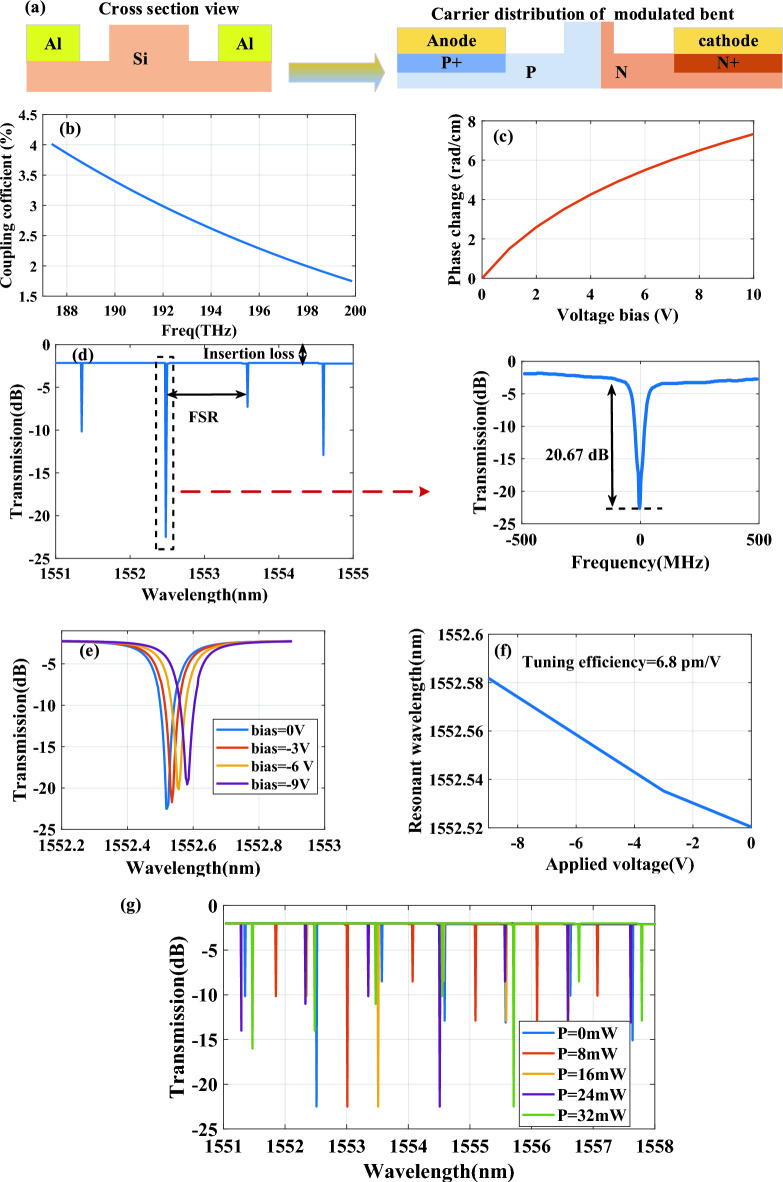
(**a**) Cross-sectional view of the carrier distribution in the PN junction. (**b**) Power coupling coefficient verses frequency for the simulated directional coupler, (**c**) Phase change as a function of applied voltage, (**d**) simulated optical transmission spectra of a MRR with a detailed zoomed-in view at zero bias voltage and zero power applied to the micro-heater, (**e**) Simulated transmission spectra of the micro ring modulator at different reverse bias voltages, (**f**) Tuning efficiency of the simulated micro ring modulator , (**g**) Transmission spectra of the ring resonator with different heating power of micro-heater.

To model the modulated bent waveguide, a PN junction is utilized, as shown in Fig. 4a. This carrier distribution acts as a phase shifter by altering the phase of the light passing through it. The application of varying bias voltages across the PN junction influences the refractive index of the waveguide's material, thereby inducing a phase shift in the light within the ring resonator.

Using 3D-FDTD simulations, we evaluated the power characteristics of the directional coupler's coupling coefficient across different frequencies, as shown in Fig. 4b. For the passive waveguide components, we employed the finite difference element (FDE) solver. Table 1 summarizes the results, displaying variations in the effective refractive index with respect to wavelength for both types of waveguides.

**Table 1:** Real and imaginary part changes of straight and passive bent waveguides refractive index in terms of wavelength.

Wavelength (um)	Refractive index straight waveguide		Refractive index passive bent waveguide	
	Real	Img ($\times {10}^{-10})$	Real	Img ($\times {10}^{-6})$
1.5	2.58854	− 5.28329	2.59173	1.56112
1.50673	2.5829	− 5.78524	2.58613	2.00191
1.51351	2.57722	− 6.28743	2.58048	2.52713
1.52036	2.57151	− 6.77335	2.5748	3.15053
1.52727	2.56576	− 7.22072	2.56909	3.88776
1.53425	2.55986	− 7.60237	2.56322	4.76946
1.54128	2.55368	− 7.87811	2.55709	5.83779
1.54839	2.54747	− 7.98835	2.55092	7.09431
1.55556	2.54122	− 7.86481	2.54471	8.56742
1.56279	2.53493	− 7.42077	2.53847	10.2891
1.57009	2.5286	− 6.54699	2.53219	12.2953
1.57746	2.52224	− 5.10674	2.52587	14.6264
1.58491	2.51583	− 2.92996	2.51952	17.3272
1.59242	2.50939	1.93733	2.51313	20.4479
1.6	2.50291	4.52324	2.50671	24.0442

Figure 4c presents a graph detailing the phase shift as a function of the applied reverse bias voltage. The performance of the proposed ring modulator was assessed using the simulated results of each component, such as the effective refractive indices and the relative phase change, to simulate a circuit as depicted in Fig. 3d.

Figure 4d illustrates the optical transmission spectra of the ring modulator without any applied electrical power to the micro-heater. The FSR, defined as the wavelength difference between the resonance dips of the ring resonator, is shown. The resonance dip with the largest extinction ratio (ER), highlighted in the black dotted box, was chosen as the notch filter for our system. The FSR is 1.023 nm, with an insertion loss of 2.18 dB and an ER of 20.67 dB. The bandwidth, calculated based on the full width at half-maximum (FWHM), is 98 MHz, indicating a high Q-factor for the ring resonator is $50.75 \times 10^{5}$.

Figure 4e displays the transmission spectra of the simulated ring modulator under four distinct reverse bias voltages applied to the RF electrodes. These spectra illustrate how varying the reverse bias voltage affects the modulator’s performance, with each voltage level corresponding to a different spectral response. This shift highlights the modulator's tunability and its ability to adjust the resonance frequency in response to the applied bias voltage. As expected, increasing the reverse bias voltage expands the depletion region within the waveguide. This reduction in the refractive index consequently shifts the resonance frequency of the ring modulator. The resonance of the ring modulator exhibits a linear shift with a tuning efficiency of 6.8 pm/V, as demonstrated in Fig. 4f. As shown in Fig. 4g, by applying electrical power to the micro-heater located above the ring resonator, the simulation results for five different applied power levels are demonstrated. It can be observed that the resonance frequency is adjustable by varying the electrical power supplied to the micro-heater.

**Figure 5:**
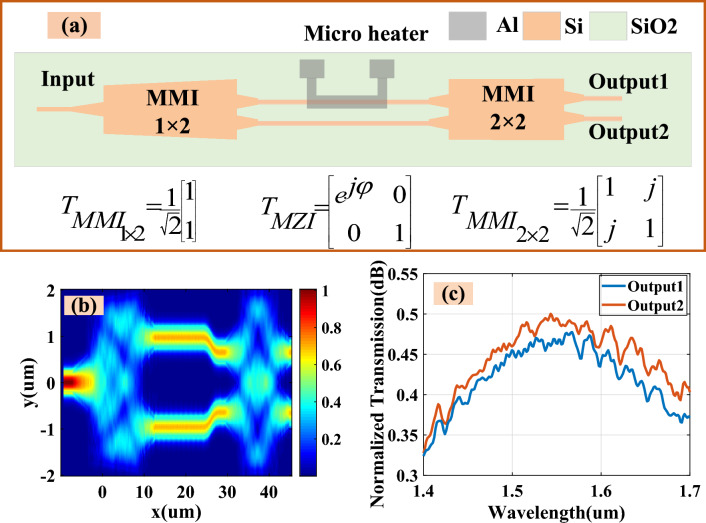
(**a**) Schematic diagram of a proposed tunable power splitter and transmission matrix of corresponding components, (**b**) field distribution in the proposed power splitter in the absence of a thermal heater, (**c**) normalized transmission verses wavelength for two outputs without thermal heater.

To simulate a tunable power spilitter, we used a configuration similar to that shown in Fig. 5a, which incorporates two MMI couplers, two identically lengthened waveguides, and a micro heater. As illustrated in Fig. 5a, the optical signal is evenly split between the two arms. A micro heater is employed to adjust the phase difference between these arms, enabling the MMI $2 \times 2$ to produce two distinct output power levels Fig. 5b shows the field distribution when there is no phase difference between the arms. As demonstrated in Fig. 5c, the normalized transmission levels for both outputs range between approximately 0.45–0.5 dB in the wavelength spectrum of 1.5–1.6 μm under zero phase difference conditions. The power ratio of these outputs can be finely controlled by altering the phase with the micro heater.

**Figure 6:**
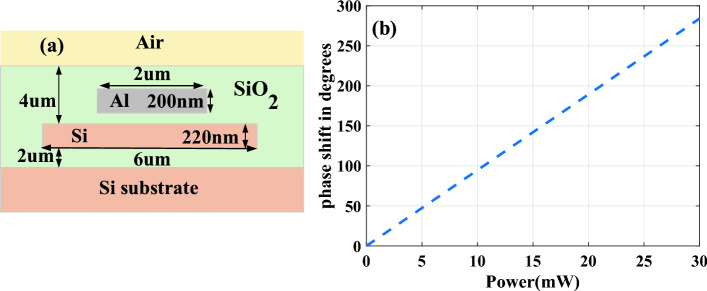
(**a**) A cross section simulation model of micro heater in the vicinity of the waveguide. (**b**) Phase shift vs applied power for the simulated micro heater.

Figure 6a depicts the cross-section of a simulated microheater adjacent to a silicon waveguide. Initially, we explore the thermal gradient induced across the waveguide by a typical heating element made of metal strips. In this setup, the waveguide measures 6 μm in width, whereas the heater is 2 μm wide and positioned 1 μm above the optical waveguide. We perform a power sweep from 0 to 30 mW to assess the device's steady-state thermal behavior within a designated heat simulation region.

Temperature profiles corresponding to varying power levels are analyzed to derive a characteristic of index perturbation, which quantifies how changes in material temperature affect its refractive index. To calculate the phase shift as per Eq. (5), we initially determine the refractive index without the index perturbation characteristic. Subsequently, incorporating the index perturbation, we calculate the refractive index across different input powers. In Fig. 6b, we plot the phase shift over a normalized length of L = 90 μm as a function of input power, based on the variations in effective index.

The setup illustrated in Fig. 5a is effectively integrated into the proposed OEO loop by manipulating the output powers. By finely adjusting the gain and loss within the system, PT-symmetry can be broken, facilitating the selection of a single frequency mode.

5 $$\Delta \varphi = \frac{{2\pi \left( {n_{eff} - n_{eff0} } \right)L}}{\lambda }$$

The transfer matrix method (TMM) is applied to the structure shown in Fig. 5a by decomposing it into three distinct components, each represented by its own transfer matrix. This method is utilized to thoroughly analyze the designed structure. The transmission function for the component through to the output is expressed as follows:

6 $$T = \frac{1}{\sqrt 2 }\left[ {\begin{array}{*{20}c} 1 & j \\ j & 1 \\ \end{array} } \right] \cdot \left[ {\begin{array}{*{20}c} {e^{j\varphi } } & 0 \\ 0 & 1 \\ \end{array} } \right] \cdot \frac{1}{\sqrt 2 }\left[ {\begin{array}{*{20}c} 1 \\ 1 \\ \end{array} } \right] = \frac{1}{2}\left[ {\begin{array}{*{20}c} {e^{j\varphi } } & j \\ {je^{j\varphi } } & 1 \\ \end{array} } \right]$$

**Figure 7:**
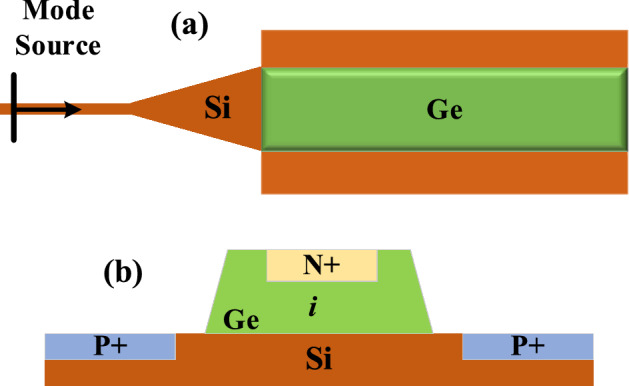
(**a**) Top view of the simulated PD, (**b**) side view of the carrier distribution in an integrated PD.

In the next phase of our study, we simulated an integrated PD, as depicted in the top-view schematic shown in Fig. 7a and the carrier distribution displayed in the cross-sectional view Fig. 7b. This PD utilizes a vertical N-I-P junction structure. For the simulation, perfectly matched layer (PML) boundary conditions were applied in all directions to minimize reflections. In this configuration, the germanium (Ge) layer serves as the absorption layer, critical for calculating the generation rate of carriers. The absorption per unit volume is quantified by evaluating the divergence of the Poynting vector. This relationship, after simplification, is expressed as follows:

7 $$P_{abs} = - 0.5real\left( {\vec{\nabla } \cdot \vec{P}} \right) = 0.5real\left( {i\omega \vec{E} \cdot \vec{D}^{*} } \right) - 0.5\omega \left| E \right|^{2} imag\left( \varepsilon \right)$$

In this context, E represents the electric field, and D represents the displacement current density. The absorption of photons per unit volume can be articulated as follows:

8 $$g = \frac{{P_{abs} }}{h\omega } = \frac{{ - 0.5\left| E \right|^{2} img\left( \varepsilon \right)}}{h}$$

**Figure 8:**
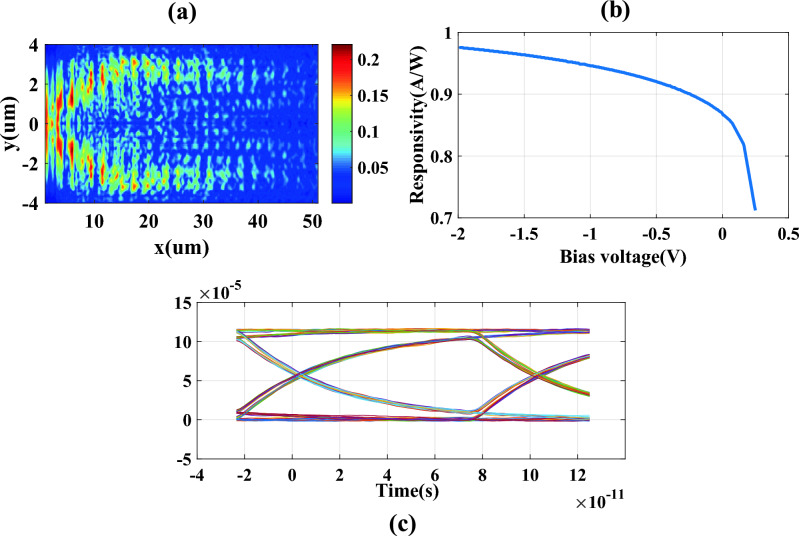
(**a**) Generation rate of simulated PD at z = 337 nm, (**b**) responsivity diagram according to voltage bias changes, (**c**) the eye diagram of the simulated PD correspond to 10 Gb/s bit rate**.**

In this formulation, h denotes the Planck constant. The absorption of photons leads to the creation of electron–hole pairs. Within the depletion zone, the electric field drives these pairs apart, generating a current flow. Figure 8a displays the generation process at a chosen cross-section along the x-direction of propagation. The plot of responsivity versus voltage bias, depicted in Fig. 8b, shows a responsivity of 0.95 A/W at a reverse voltage of − 1 V. The dark current for the PD is determined to be $0.35\mu A$ at a temperature of 300 K, with a bandwidth of approximately 4 GHz at the same reverse bias voltage. Utilizing all the results from previous steps, we develop a compact model of the simulated detector for integration into the proposed OEO loop circuit. Figure 8c illustrates the eye diagram of the detector, achieving a bit rate of 10 Gb/s.

### Simulated proposed OEO

Figure 1 presents the proposed OEO. To evaluate its performance, we integrate the characteristics of each simulated component into the circuit model. These characteristics, which include S-parameters, effective indices, dark current, and responsivity, have been determined in previous steps and will be utilized in the circuit model. The structure is designed to effectively break PT-symmetry by adjusting the bias voltage applied to the microheater, which controls the gain and loss within the system. This adjustment enables the selection of a single frequency mode, eliminating the need for a narrowband microwave filter. The gain and loss coefficients can be expressed as follows (see Supplementary Material).

9 $$g = \frac{{\ln \left| {g_{{{\text{i}},\max }} (1 + \sin \varphi )} \right|}}{{\tau_{r} }}$$

10 $$\alpha = \frac{{\ln \left| {g_{{{\text{i}},\max }} (1 - \sin \varphi )} \right|}}{{\tau_{r} }}$$

In this setup, $g_{i,\max }$ signifies the maximum round-trip gain within the proposed OEO loop.$\theta$ indicates the polarization angle, and $\tau_{r}$ denotes the round-trip path of the OEO. According to the relationships outlined, the angle $\theta$ needs to be adjusted to a value that fulfills the PT-symmetry condition ($g = - \alpha$).

11 $$\theta = \pm \arccos \left( {\frac{1}{{g_{i,\max } }}} \right)$$

**Figure 9:**
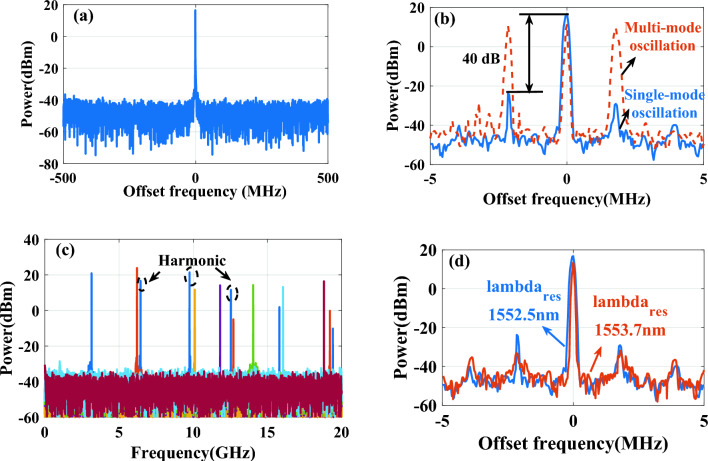
The electrical spectrum of the generated microwave signals with at a central frequency of 11.5 GHz (**a**) The oscillation was measured in single-mode with a span of 1 GHz and a RBW of 3 MHz, (**b**) The electrical spectra of single‐mode oscillation (blue solid curve) and multimode oscillation (red dashed curve) with a span of 10 MHz and an RBW of 50 kHz, (**c**) Frequency tunability of the proposed PT-symmetric OEO, (**d**) Comparison of the OEO performance results between two resonance wavelengths (1552.5 nm and 1553.7 nm) for a tunable high Q-factor MRR.

PT symmetry can be maintained by adjusting the phase shift in one arm of the Mach–Zehnder Interferometer (MZI) when $g_{i,\max }$ exceeds 1. This study involved circuit modeling and fine-tuning to analyze the OEO loop. The electrical spectrum of the proposed OEO, with 1.6 V applied to the microheater, is displayed in Fig. 9a. This figure illustrates the electrical spectra of the signal at a frequency of 11.5 GHz, with an offset frequency of 1 GHz and a resolution bandwidth (RBW) of 3 MHz. Figure 9b shows the comparison between the electrical spectra of single-mode and multimode oscillations with a span of 10 MHz and an RBWof 50 kHz. The SMSR of this signal is 40 dB. It is evident that the single-mode frequency has been achieved in the proposed structure without the need to use a narrow-band microwave filter, simply by leveraging the capability to break PT symmetry. It is important to note that the oscillation frequency can be adjusted by tuning the center frequency of the MPF. In this design, the center frequency of the generated microwave signal can be varied across a wide range by controlling the phase through the microheater and adjusting the laser wavelength, though this range is limited by the FSR of the MRR. The oscillation frequency can be varied from 0 to 20 GHz by changing the laser wavelength, with the tuning range restricted by the FSR of the MRR. Especially, the high-order harmonics can be observed in Fig. 9c. Nonlinearity in the OEO loop is the primary source of high-order harmonics^5,10^. In our proposed PT-symmetric OEO, these high-order harmonics are mainly generated by the electrical amplifier and the multiple test ports used to monitor optical power during simulations. However, these test ports can be removed in practical applications, which would reduce the power of high-order harmonics. Additionally, using devices with lower nonlinear efficiencies can further decrease the power of these high-order harmonics. In Fig. 9d, we compare the performance of proposed OEO using a tunable MRR and tuning by microheater at two specific resonance wavelengths: 1552.5 nm and 1553.7 nm. The performance metrics evaluated is the output power spectrum. As observed, the OEO at both wavelengths has a similar spectrum with a main resonance peak at the corresponding frequencies. The peak shifts slightly between 1552.5 nm and 1553.7 nm, reflecting the change in resonance frequency due to wavelength tuning. At both wavelengths, the sidebands around the resonance peak are well suppressed, indicating minimal unwanted frequencies and harmonics. This is because the high quality Q-factor of the MRR results in a narrow and sharp resonance peak, allowing for precise selection of the desired frequency. Overall, while the exact position of the resonance peak changes with the wavelength, the overall spectral response remains consistent, demonstrating the tunability and stable performance of the OEO across different wavelengths.

### Phase noise of the proposed OEO

Generally, the OEO structure displays inherent nonlinearity, but a linear model is sufficient to accurately depict its phase noise when there are small phase perturbations to the stable oscillation. Several studies have investigated the behavior of OEO devices by exploring the impact of effective noise sources or under particular conditions^28–30^.

**Figure 10:**
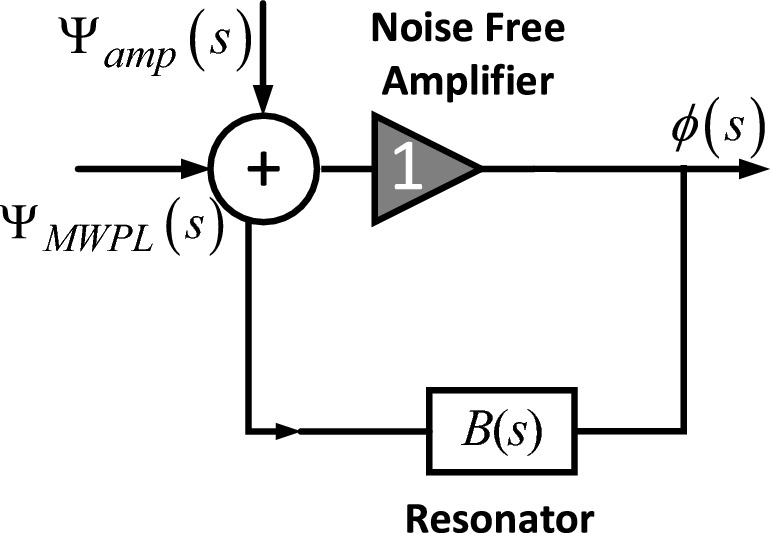
Modeling the proposed PT-symmetric OEO using phase-space analysis.

The phase noise model for the PT-symmetry OEO under discussion is depicted in Fig. 10. This model comprises an amplifier and a microwave photonic link (MWPL), each characterized by their respective equivalent input noises, represented as $\psi_{MWPL}$ and $\psi_{amp}$, respectively. Additionally, the model includes a feedback loop with a resonator that possesses a phase transfer function of $B(s)$. In this specific setup, the amplifier is set to a gain of one, which means it transmits the input phase unaltered.

Based on this model, the output phase noise can be described through the application of linear feedback theory^31–33^.

12 $$L(\omega ) = 10\log \left( {\frac{{S_{\varphi } \left( \omega \right)}}{2}} \right) = 10\log \left( {\frac{{\left[ {\left| {H_{1} (j\omega )} \right|^{2} S_{{\psi_{amp} }} \left( \omega \right) + \left| {H_{2} (j\omega )} \right|^{2} S_{{\psi_{MWPL} }} \left( \omega \right)} \right]}}{2}} \right)$$

The power spectral densities (PSD) of $S_{{\psi_{amp} }} (\omega )$ and $S_{{\psi_{MWPL} }} (\omega )$, respectively, at an offset $\Delta \omega$ from the carrier frequency, can be represented. Additionally, the phase transfer functions of these two noise sources,$H_{1} (j\omega )$ and $H_{2} (j\omega )$, can be written as

13 $$H(j\omega ) = H_{1} (j\omega ) = H_{2} (j\omega ) = \frac{1}{1 - B(j\omega )}$$

The transfer function can be expressed as

14 $$B(j\omega ) = \frac{1}{1 + j\omega \tau }$$

By inserting Eq. (14) into Eq. (13), the square of the phase transfer function can be expressed as

15 $$\left| {H(j\omega )} \right|^{2} = \frac{{\tau^{2} \omega^{2} + 1}}{{\tau^{2} \omega^{2} }} = 1 + \frac{1}{{\tau^{2} \omega^{2} }}$$

Therefore, by combining (12) and (15), the output phase noise can be written as

16 $$L(\omega ) = 10\log \left( {\frac{{S_{\varphi } \left( \omega \right)}}{2}} \right) = 10\log \left( {\frac{{[1 + \frac{1}{{\omega^{2} \tau^{2} }}]\left[ {S_{{\psi_{amp} }} \left( \omega \right) + S_{{\psi_{MWPL} }} \left( \omega \right)} \right]}}{2}} \right)$$

Recalling that $$\tau = {\raise0.7ex\hbox{${2Q}$} \!\mathord{\left/ {\vphantom {{2Q} {\omega_{0} }}}\right.\kern-0pt} \!\lower0.7ex\hbox{${\omega_{0} }$}} = {\raise0.7ex\hbox{$Q$} \!\mathord{\left/ {\vphantom {Q {\pi f_{0} }}}\right.\kern-0pt} \!\lower0.7ex\hbox{${\pi f_{0} }$}}$$, and using $$\omega = 2\pi f$$ we get

17 $$L(f) = 10\log \left( {\frac{{\left[ {1 + \frac{1}{{f^{2} }}\left( {\frac{{f_{0}^{2} }}{2Q}} \right)} \right]\left[ {S_{{\psi_{amp} }} \left( f \right) + S_{{\psi_{MWPL} }} \left( f \right)} \right]}}{2}} \right)$$

The primary sources of noise impacting the MWPL within the OEO loop are relative intensity noise (RIN), shot noise, and thermal noise. Consequently, $S_{{\psi_{MWPL} }} (f)$ can be characterized as follows^34–36^:

18 $$S_{{\psi_{MWPL} }} \left( f \right) = \frac{{\left( {k_{B} T_{0} + \left\langle {I_{D} } \right\rangle^{2} N_{rin} R_{0} + 2e\left\langle {I_{D} } \right\rangle R + G\alpha k_{B} T_{0} } \right)}}{{P_{RF} }}$$

The output oscillation power is denoted by $P_{RF}$, and the PSD of the photodetector's thermal noise is represented by $k_{B} T_{0}$, where $k_{B} = 1.38 \times 10^{ - 23} {J \mathord{\left/ {\vphantom {J {^\circ K}}} \right. \kern-0pt} {^\circ K}}$ is Boltzmann’s constant and $T_{0} = 290K$ is the ambient temperature. $\left\langle {I_{D} } \right\rangle^{2} N_{rin} R$ represents the PSD of the laser RIN, $N_{rin}$ is the RIN of the laser light; where *R* is the resistance of the photodetector's load impedance. $2e\left\langle {I_{D} } \right\rangle R$ represents the PSD of the shot noise in the photodetector. The PSD of the thermal noise from the ring modulator at the output of the MWPL is denoted by $G\alpha k_{B} T_{0}$. The variable $G$ signifies the gain of the MWPL, and the constant $\alpha$ measures the influence of the ring modulator's thermal noise on the MWPL's output^30^.

Additionally, the noise sources in the electrical components can be divided into two categories: thermal noise and flicker noise. Consequently, $S_{{\psi_{amp} }} (\omega )$ can be formulated as follows:

19 $$S_{{\psi_{amp} }} (\omega ) = b_{0} + \frac{{b_{ - 1} }}{f}$$

Here, $b_{0} = {{Fk_{B} T_{0} } \mathord{\left/ {\vphantom {{Fk_{B} T_{0} } {P_{RF} }}} \right. \kern-0pt} {P_{RF} }}$ represents the white noise of the amplifier,$F$ denotes its noise factor, and the constant $b_{ - 1}$ corresponds to the flicker noise of the amplifier, measured at an offset frequency of 1 Hz from the carrier signal.

**Figure 11:**
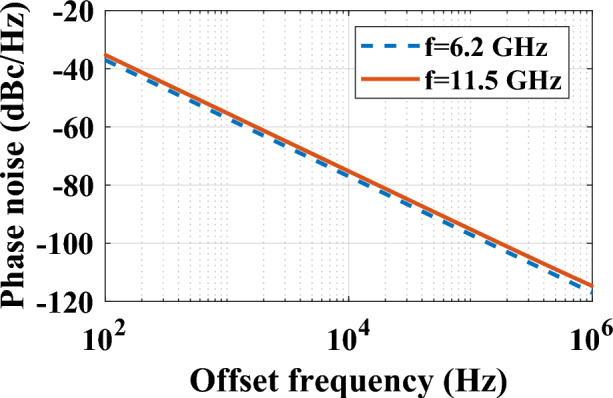
Phase noise calculated for the proposed PT-symmetric OEO when the oscillation frequencies are 6.2 GHz (blue dashed curve) and 11.5 GHz (red solid curve), respectively.

In this study, the amplitude of the output microwave signal is set at 1.8 V. With a load resistance (R) of 50 Ω, the output power will be $P_{RF} = 49mW$. In addition, the average PD current is computed $\left\langle {I_{D} } \right\rangle = 135mA$ and $N_{rin} = - 165dBc/Hz$ taken into account for the TLS laser. Figure 11 illustrates the phase noise spectrum for the proposed PT-symmetry OEO, utilizing the typical values specified in Eqs. (17)–(19). The figure shows the calculated phase noise of the microwave signals generated at 6.2 GHz (blue dashed curve) and 11.5 GHz (red solid curve). As observed, the respective single-sideband (SSB) phase noise at a 10 kHz offset frequency is − 79.22 dBc/Hz and − 76.5 dBc/Hz.

### Analysis of instabilities

Typically OEOs face various instabilities, such as mode hopping, which can adversely affect signal quality and stability. Mode hopping occurs when the oscillation frequency shifts between different modes, leading to reduced stability and increased phase noise. In our design, PT symmetry breaking is employed as a robust mode selection mechanism to achieve stable single-mode oscillation. By carefully controlling the gain and loss coefficients using a tunable MZI, we effectively suppress unwanted modes and mitigate mode hopping.

The coupled differential equations governing the oscillation modes were solved to analyze the transient and steady-state behavior of the OEO under various operating conditions. The simulation result demonstrates that PT symmetry breaking significantly enhances mode stability by increasing the gain difference between the dominant oscillation mode and other modes. This approach ensures that only the desired mode reaches oscillation, thereby improving the overall stability and performance of the OEO system.

Simulation results demonstrate that precise adjustment of the voltage applied to the microheater can achieve stable single-mode oscillation. These results are consistent with simulated data obtained under different operating conditions. For example, at an oscillation frequency of 11.5 GHz, the SMSR reaches 40 dB, indicating stable system performance.

One of the significant factors that may cause frequency shift in reality is the drift in the MRR resonant wavelength, which occurs due to environmental perturbations and thermal cross-talk. To reduce its impact, thermal isolation can be implemented around the MRR. Additionally, the devices can be placed in a temperature-controlled environment to compensate for the effects of environmental perturbations. Moreover, thermoelectric coolers with a feedback loop can be used for long-term stability.

## Conclusion

In this study, we present and simulate a tunable integrated PT-symmetric OEO. This design utilizes the concept of PT-symmetry breaking by incorporating a ring modulator that functions both as a modulator and a resonator to selectively determine the frequency mode. The system includes a MRR with a high Q-factor, an adjustable power splitter, and a photodetector, all of which are integrated into the system. The performance of each component was verified through simulations. The proposed OEO's effectiveness was evaluated based on simulation results and theoretical analysis, focusing on the frequency spectrum and phase noise characteristics of the generated microwave signal. The results demonstrate that the phase noise for the 6.2 GHz and 11.5 GHz signals stand at − 79.22 dBc/Hz and − 76.5 dBc/Hz at a 10 kHz offset frequency, and the SMSR is 40 dB. This innovative setup provides a practical approach for achieving a single frequency mode without relying on a narrowband filter and is suitable for integrated implementation.

### Supplementary Information

Supplementary Information.

## Data Availability

The calculated results during the current study are available from the corresponding author on reasonable request.
