# Supplementary material for: Design and simulation of a tunable parity-time symmetric optoelectronic oscillator utilizing integrated components
Source: Sci Rep. 2024 Jul 11;14:16014. doi: 10.1038/s41598-024-67047-0 (PMC11239854; doi:10.1038/s41598-024-67047-0)
Supplement: Supplementary file 1 — Supplementary Information. [file 41598_2024_67047_MOESM1_ESM.docx]

Online supporting information for the following article

Design and Simulation of a Tunable Parity-Time Symmetric Optoelectronic Oscillator Utilizing Integrated Components

Farnaz Ahmadfard ^1 *^ and S. Esmail hosseini^1^

^1^ Department of Communications and Electronics, School of Electrical and Computer Engineering, Shiraz University, Shiraz, Iran

**Email:* [*f.ahmadfard@shirazu.ac.ir*](mailto:f.ahmadfard@shirazu.ac.ir)

Here we analyze the proposed structure of OEO Fig.1. It is evident that light with a specific frequency is transmitted through micro ring, which serves as both a resonator and a modulator. Initially, the output of the modulated signal can be represented as follows:

| $E_{PM}(t)=E_{0}\mathrm{Re}\{\exp(\mathrm{j}2\pi f_{c}t)\exp(\mathrm{j}\beta\cos(2\pi f_{m}t))\}$ | (S1) |
| --- | --- |

The variables $E_{0}$, $\beta$, and $f_{m}$ represent the amplitude of the optical carrier, the modulation index, and the frequency of the microwave signal, respectively. For the small signal, Eq. (S1) can be represented as:

| $E_{PM}(t)=E_{0}\mathrm{Re}\{J_{0}(\beta)\exp(\mathrm{j}2\pi f_{c}t)+J_{1}(\beta)\exp(\mathrm{j}2\pi(f_{c}+f_{m})t)-J_{1}(\beta)\exp(\mathrm{j}2\pi(f_{c}-f_{m})t)\}$ | (S2) |
| --- | --- |

Here,refers to the Bessel function of the first kind of order n. Additionally, considering that the MRR functions as a notch filter, by tuning its resonance frequency using the micro-heater to align with $f_{c}+f_{m}$, the desired filtering effect is achieved. Based on the structure depicted in Fig. 1, it is clear that the modulated signal will go through the PC2. In this case, the PC2 can adjust the polarization angle. Therefore, the optical signals at the output of PC2 can be represented as

|  | (S3) |
| --- | --- |

Where $T(f)$and are the amplitude and phase of the MRR at a given frequency, respectively. Considering that the MRR acts as a notch filter in the +1^st^ side band, the amplitude of the transmission at this frequency is zero ($\left|T(f_{c}+f_{m})\right|=0$). Thus, Eq. (S3) can be simplified as follows:

| $E_{MRR}(t)=E_{0}\mathrm{Re}\{J_{0}(\beta)\exp(\mathrm{j}2\pi f_{c}t)-J_{1}(\beta)\exp(\mathrm{j}2\pi(f_{c}-f_{m})t)\}$ | (S4) |
| --- | --- |

After this, based on transfer matrix Eq.(6), the two resulting optical fields can be obtained and represented as:

|  | (S5) |
| --- | --- |

Here, represents the phase shift of the optical signal in the upper arm of the mode selector and can be tuned by a microheater. Afterwards, the two optical signals are directed towards two identical PDs to convert them into microwave signals. The amplified electrical output can be expressed as:

|  | (S6) |
| --- | --- |

The gain of the electrical amplifier is denoted by $\mathrm{G}_{A}$, the responsivity of the PD is represented by $\rho$, the load resistance is indicated by $R$, and the overall electrical loss of the loop is denoted by $T_{ff}$. However, it is known that the round-trip voltage gain can be expressed as:

| $G=\frac{V_{out}(t)}{V_{in}(t)}$ | (S7) |
| --- | --- |

Eq. (S7) can be used to calculate the voltage gain of the two coupled loops as follows:

|  | (S8) |
| --- | --- |
| $g_{\mathrm{i},\max}=\frac{\mathrm{G}_{A}\left\vert E_{0}\right\vert^{2}J_{0}(\beta)J_{1}(\beta)\rho RT_{ff}}{V_{m}}$ | (S9) |

Where $g_{\mathrm{i},\max}$ denotes the maximum possible voltage gain achieved in the proposed integrated PT-symmetric OEO. Alternatively, the relationship relating to the round-trip voltage gain can be represented as follows:

| $G=\frac{V(t+\tau)}{V(t)}=\frac{V_{0}\mathrm{e}^{j\omega_{m}t}\mathrm{e}^{g(\mathrm{t}+\tau_{r})}}{V_{0}\mathrm{e}^{j\omega_{m}t}\mathrm{e}^{g\mathrm{t}}}=\mathrm{e}^{g\tau_{r}}$ | (S10) |
| --- | --- |

According to Eq. (S10), the gain coefficient ($g$) can be stated in terms of the round-trip time () as follows:

| $g=\frac{\ln\left\vert G\right\vert}{\tau_{r}}$ | (S11) |
| --- | --- |

Therefore, the gain/loss coefficients of the two coupled loops can be stated using Eqs. (S8) and (S11) in the following manner:

|  | (S12) |
| --- | --- |
|  | (S13) |
